# Supplementary material for: Impact of Chronic Kidney Disease and Dialysis on Outcome after Surgery for Infective Endocarditis
Source: J Clin Med. 2023 Sep 13;12(18):5948. doi: 10.3390/jcm12185948 (PMC10532068; doi:10.3390/jcm12185948)
Supplement: Supplementary file 1 [file jcm-12-05948-s001.zip › jcm-2564872-supplementary.pdf]

Table S1: Predisposing factors for Infective Endocarditis.

**Variables** [a]	**All patients** n **= 592**	**Non-HD group** n **= 534**	**HD group** n **= 58**	***p*-value**
History of IE	36 (6.1%)	31 (5.8%)	5 (8.6%)	0.422
Previous surgery for IE	34 (5.9%)	31 (5.9%)	3 (5.5%)	0.886
Valvular heart disease	185 (31.4%)	166 (31.2%)	19 (32.8%)	0.808
Mitral valve prolapse	31 (5.3%)	31 (5.8%)	0 (0.0%)	**0.010**
Vascular graft implantation	11 (1.9%)	8 (1.5%)	3 (5.2%)	0.097
Previous coronary angiography	58 (34.7%)	45 (31.0%)	13 (59.1%)	**0.010**
Congenital heart disease	15 (2.5%)	14 (2.6%)	1 (1.7%)	0.660
Cyanotic heart disease	2 (0.3%)	2 (0.4%)	0 (0.0%)	0.520
Immunosuppression	12 (2.0%)	8 (1.5%)	4 (6.9%)	**0.024**
HIV Infection	13 (2.2%)	13 (2.4%)	0 (0.0%)	0.099
Active Malignancy	53 (9.0%)	47 (8.8%)	6 (10.3%)	0.702
Current Alcohol abuse	53 (9.0%)	46 (8.6%)	7 (12.1%)	0.387
Current intravenous drug abuse	37 (6.3%)	35 (6.6%)	2 (3.4%)	0.314
Active Hepatitis	35 (5.9%)	33 (6.2%)	2 (3.4%)	0.366
Hepatitis B Infection	5 (0.8%)	3 (0.6%)	2 (3.4%)	0.073
Hepatitis C Infection	33 (5.6%)	33 (6.2%)	0 (0.0%)	**0.008**

For listed nominal variables[a], the absolute number, n, is calculated with percentage (%). **Bold** indicates $P < 0.05$.

Non-HD group: patients without Haemodialysis preoperatively, HD group: patients with Haemodialysis preoperatively, IE: infective endocarditis, HIV: human immunodeficiency virus.

Table S2: Distribution of IE-affected valves in patients with and without preoperative dialysis

**Variables** [a]	**All patients** ***n* = 592**	**Non-HD group** ***n* = 534**	**HD group** ***n* = 58**	***p*-value**
Left heart IE	556 (94.2%)	502 (94.4%)	53 (93.1%)	0.704
Right heart IE	40 (6.7%)	36 (6.8%)	4 (5.9%)	0.970
Aortic valve IE	347 (58.8%)	320 (60.2%)	27 (46.5%)	**0.046**
Mitral valve IE	282 (47.7%)	246 (46.2%)	36 (62.0%)	**0.022**
Tricuspid valve IE	37 (6.2%)	33 (6.2%)	4 (6.9%)	0.836
Pulmonary valve IE	3 (0.8%)	3 (0.6%)	0 (0.0%)	0.566
Double valve IE	83 (14.0%)	72 (13.5%)	11 (19.0%)	0.259
Triple valve IE	1 (0.1%)	1 (0.2%)	0 (0.0%)	0.741
NVE	448 (77.6%)	407 (78.0%)	41 (74.5%)	0.562
PVE	129 (22.3%)	115 (22.0%)	14 (25.5%)	0.562
Aortic valve prosthesis	100 (16.9%)	93 (17.5%)	7 (12.1%)	0.297
Mitral valve prosthesis	27 (4.5%)	20 (3.8%)	7 (12.1%)	**0.014**
Tricuspid valve prosthesis	0 (0.0%)	0 (0.0%)	0 (0.0%)	
Pulmonary valve prosthesis	2 (0.3%)	2 (0.4%)	0 (0.0%)	0.520

For listed nominal variables [a], the absolute number, n, is calculated with percentage (%). **Bold** indicates $P < 0.05$.

Non-HD group: patients without Haemodialysis preoperatively. HD-group: patients with Haemodialysis preoperatively.

IE: infective endocarditis, NVE: IE of a native valve, PVE: IE of a prosthetic valve.

Table S3: Microbiological findings in patients with and without preoperative dialysis

**Variables** [a]	**All patients** *n* **= 592**	**Non-HD group** *n* **= 534**	**HD group** *n* **= 58**	***p*-value**
Staphylococcus aureus	147 (24.9%)	124 (23.3%)	23 (39.7%)	**0.006**
CoNS	75 (12.7%)	69 (13.0%)	6 (10.3%)	0.569
Streptococcus pyogenes	15 (9.6%)	13 (2.4%)	2 (3.4%)	0.659
Streptococcus viridans	106 (17.9%)	99 (18.6%)	7 (12.1%)	0.218
Other Streptococci	43 (7.2%)	42 (7.9%)	1 (1.7%)	**0.044**
Enterococci	84 (14.2%)	79 (14.8%)	5 (8.6%)	0.197
Gram-negative HACEK	5 (0.8%)	4 (0.8%)	1 (1.7%)	0.494
Gram-negative non-HACEK	21 (3.5%)	16 (3.0%)	5 (8.6%)	0.057
Fungi	8 (1.3%)	7 (1.3%)	1 (1.7%)	0.806
Other Organisms	55 (9.3%)	53 (8.9%)	3 (5.2%)	0.397
No organisms detected	88 (14.9%)	79 (14.8%)	9 (15.5%)	0.892
Multiple infections	57 (9.6%)	52 (9.7%)	5 (8.6%)	0.723

For listed nominal variables [a], the absolute number, n, is calculated with percentage (%). **Bold** indicates $P < 0.05$.

Non-HD group: patients without Haemodialysis preoperatively. HD-group: patients with Haemodialysis preoperatively. IE: infective endocarditis, CoNS: coagulase-negative staphylococci. **HACEK**: **H**aemophilus species, **A**ggregatibacter species, **C**ardiobacterium hominis, **E**ikenella corrodens and **K**ingella species.

Table S4: Operative data in surgically treated infective endocarditis patients

**Variables**	**All patients** *n* **= 592**	**Non-HD group** *n* **= 534**	**HD group** *n* **= 58**	***p*-value**
Abscess	175 (29.6%)	157 (29.5%)	18 (31.0%)	0.809
Fistula	15 (2.5%)	13 (2.4%)	2 (3.4%)	0.659
Perforation	112 (18.9%)	100 (18.8%)	12 (20.7%)	0.727
Operation time (minutes)	205.0 [160.0–264.0][a]	202.0 [158.0–260.0][a]	246.0 [183.7–283.0] [a]	**0.009**
CPB time (minutes)	115.0 [87.0 –155.0][a]	114.0 [86.0–152.5][a]	140 [103.7 –182.7] [a]	**0.003**
Cross-clamp time (minutes)	76.0 [57.0–100.0][a]	74.0 [56.0–58.0][a]	96.0 [64.0–126.7][a]	**<0.001**

For nominal variables, the absolute number (n) is calculated with percentage (%). Metric, non-normally distributed, variables are calculated as median with 25th and 75th percentiles [[a]]. **Bold** indicates $p<0.05$.

Non-HD group: patients without Haemodialysis preoperatively, HD group: patients with Haemodialysis preoperatively, CPB-time: cardiopulmonary bypass time.

Table S5: Causes of death in surgically treated infective endocarditis patients.

**Variables** [a]	**All patients** **n = 592**	**Non-HD group** **n = 534**	**HD group** **n = 58**	***p*-value**
Septic shock	52 (8.8%)	37 (7.0%)	15 (25.9%)	**<0.001**
Multiorgan failure	29 (4.9%)	23 (4.3%)	6 (10.3%)	0.072
Cardiogenic shock	16 (2.7%)	14 (2.6%)	2 (3.4%)	0.726
Right-sided heart failure	1 (0.16%)	1 (0.2%)	0 (0.0%)	0.649
Myocardial infraction	2 (0.33%)	1 (0.2%)	1 (1.7%)	0.149
Pericardial tamponade	1 (0.16%)	0 (0.0%)	1 (1.7%)	0.031
Respiratory failure	11 (1.8%)	11 (2.1%)	0 (0.0%)	0.129
Mesenterial ischemia	6 (1.0%)	5 (0.9%)	1 (1.7%)	0.603
Ischemic stroke	2 (0.34%)	2 (0.4%)	0 (0.0%)	0.520
Intracranial haemorrhage	8 (1.3%)	5 (0.9%)	3 (5.2%)	0.035
Perioperative bleeding	6 (1.0%)	5 (0.9%)	1 (1.7%)	0.603
Gastrointestinal bleeding	1 (0.16%)	1 (0.2%)	0 (0.0%)	0.649
Other bleeding	3 (0.5%)	2 (0.4%)	1 (1.7%)	0.265
Malignancy	3 (0.5%)	3 (0.6%)	0 (0.0%)	0.430
IE recurrence	5 (0.8%)	5 (0.9%)	0 (0.0%)	0.308
AKI	1 (0.16%)	1 (0.2%)	0 (0.0%)	0.649
Others	6 (1.0%)	6 (1.1%)	0 (0.0%)	0.264

For listed nominal variables [a], the absolute number, n, is calculated with percentage (%). **Bold** indicates p<0.05. Non-HD group: patients without Haemodialysis preoperatively. HD group: patients with Haemodialysis preoperatively. IE: infective endocarditis, AKI: acute kidney injury.

Table S6: Survival according to the underlying stage of CKD

	**All patients** *n* = 530	**G1 patients** *n* = 143	**G2 / G3a** *n* = 228	**G3b / G4** *n* = 139	**G5 patients** *n* = 22	***p*-value**
30-day mortality	74/389 (19.0%)	5/89 (5.6%)	28/177 (15.8%)	34/110 (30.9%)	7/13 (53.8%)	**<0.001**
1-year mortality	118/366 (32.2%)	13/84 (15.5%)	46/163 (28.2%)	50/107 (46.7%)	9/12 (75.0%)	**<0.001**
Median survival (years)	4.4 [4.0–4.6][a]	5.3 [4.7–5.9][a]	4.5 [4.1–5.0][a]	3.2 [2.6–3.8][a]	0.7 [0.3–1.3][a]	**<0.001**

For nominal variables, the absolute number, n, is calculated with percentage (%). Metric, non-normally distributed, variables are calculated as median with 25th and 75th percentiles [[a]]. **Bold** indicates p<0.05.

CKD: chronic kidney disease**,** G1 group = patients without CKD, G2 / G3a group = patients with mild to moderate, G3b / G4 group = patients with moderate to severe CKD.
